# Supplementary material for: Effects of the Density of Invasive Lantana camara Plants on the Biodiversity of Large and Small Mammals in the Groenkloof Nature Reserve (GNR) in South Africa
Source: Biology (Basel). 2023 Feb 13;12(2):296. doi: 10.3390/biology12020296 (PMC9953020; doi:10.3390/biology12020296)
Supplement: Supplementary file 1 [file biology-12-00296-s001.zip › Supplementary Table S1.pdf]

Table S1: Number of tracks of large mammal species and the treatments where the species were recorded inside the Groenkloof Nature Reserve, South Africa. See methodology for treatments definition.

**Species**	**Foraging guilds**	**Treatments**						
		**control**	***ca. 2 yrs***	**ca.10 yrs**	**ca.20 yrs**	***cl.<2 yrs***	***cl.3-5 yrs***	**Total**
Equus quagga (Boddaert, 1785)	Grazer	16	41	24	6	20	13	120
Canis moesomelas (von Schreber, 1775)	Carnivore	23	5	5	1	5	4	43
Sylvicapra grimmia (Linnaeus, 1758)	Browser	13	16	0	0	35	10	74
*Tragelaphus strepsiceros (*Pallas, 1766*)*	Browser	9	22	10	0	0	31	72
Aepyceros melampus (Sundevall, 1847)	Mixed feeder	3	0	21	0	10	0	34
Connochaetes taurinus (Burchell, 1823)	Grazer	44	10	12	0	11	13	90
Alcelaphus buselaphus (Pallas, 1766)	Grazer	15	0	8	61	0	12	96
*Giraffa camelopardalis (Briss*on, 1752)	Browser	14	30	13	12	36	36	141
Hippotragus niger (Harris, 1838)	Grazers	26	0	15	8	29	29	107
*Equus caballus (*Linnaeus, 1758)	Grazers	23	10	0	8	4	7	52
**Species Abundance**		186	134	108	96	150	155	829
**Species richness index**								
menhinick		0.769	0.653	0.612	0.604	0.722	0.733	
**Species diversity indices**								
Shannon		2.140	1.850	1.757	1.183	1.981	1.999	

Simpson	0.866	0.844	0.802	0.562	0.821	0.850
**Species Evenness**						
Pielou evenness Shannon	0.952	0.910	0.903	0.660	0.889	0.929
Pielou evenness Simpson	0.412	0.394	0.376	0.314	0.384	0.408

Species abundance=the total abundance of individual present (per area)
Species richness index (Menhinick's)= the number of species (n) divided by the square-root of the total number of individuals (N): Take account sample size
Species diversity indices (Shannon and Simpson) =Considers both species richness as well as the dominance/evenness of the species.
Species evenness=Is the measure of how even a community or ecosystem is in terms of abundance of its species
