# Supplementary material for: Effects of the Density of Invasive Lantana camara Plants on the Biodiversity of Large and Small Mammals in the Groenkloof Nature Reserve (GNR) in South Africa
Source: Biology (Basel). 2023 Feb 13;12(2):296. doi: 10.3390/biology12020296 (PMC9953020; doi:10.3390/biology12020296)
Supplement: Supplementary file 1 [file biology-12-00296-s001.zip › Supplementary Table S2.pdf]

Supplementary Table S2: Abundance and species richness of rodents in six treatments inside the Groenkloof Nature Reserve, in South Africa surveyed in 2019 and 2020. See methodology for description of treatments.

**Species**	**ca 2 yrs**	**ca 10 yrs**	**ca 20yrs**	**cl<2yrs**	**cl 3-5 yrs**	**control**	**Total**
**Order: Rodentia**							
Aethomys sp.	2	0	1	0	1	1	5
Saccostomus campestris	0	1	0	0	1	4	6
Rhabdomys pumilio	7	1	3	7	9	11	38
Lemniscomys rosalia	2	3	0	2	2	1	10
Mastomys coucha	0	2	1	1	1	2	7
Total number of individuals	11	7	5	10	14	19	66
Estimated richness Chao 1	4.0 ± 0.81	3.0 ± 0.2	2.0 ± 0.3	5.0 ± 0.42	6.0 ± 0.16	9.0 ± 3.7	
%completeness of sample	80	68	78	73	87	76	
Estimated richness Jack1	5.1 ± 1.54	4.8 ± 1.03	4.7 ± 1.01	6.1 ± 1.54	6.5 ± 0.70	9.7 ± 1.52	
%completeness of sample	80	90	90	90	87	98	
Total trap Success	0,003	0,002	0,001	0,003	0,004	0,004	
**Diversity indices**							
Simpson	0.528	0.698	0.56	0.551	0.46	0.603	
Shannon	0.907	1.277	0.95	1.127	0.801	1.913	
Margalef's	0.904	1.511	1.341	1.336	0.948	1.147	

Trap Success (Total catch x 100) / Trap nights)
